# Endophytic *Bacillus altitudinis* Strain Uses Different Novelty Molecular Pathways to Enhance Plant Growth

**DOI:** 10.3389/fmicb.2021.692313

**Published:** 2021-06-25

**Authors:** Dening Zhang, Hongli Xu, Jingyao Gao, Roxana Portieles, Lihua Du, Xiangyou Gao, Carlos Borroto Nordelo, Orlando Borrás-Hidalgo

**Affiliations:** ^1^Joint R&D Center of Biotechnology, Retda, Yota Bio-Engineering Co., Ltd., Rizhao, China; ^2^VBS Biotec SA, Mexico, Mexico; ^3^State Key Laboratory of Biobased Material and Green Papermaking, Shandong Provincial Key Lab of Microbial Engineering, Qilu University of Technology (Shandong Academy of Science), Jinan, China

**Keywords:** endophytic bacteria, *Bacillus altitudinis*, plant growth, transcriptome, *Glyceria chinensis*

## Abstract

The identification and use of endophytic bacteria capable of triggering plant growth is an important aim in sustainable agriculture. In nature, plants live in alliance with multiple plant growth-promoting endophytic microorganisms. In the current study, we isolated and identified a new endophytic bacterium from a wild plant species *Glyceria chinensis* (Keng). The bacterium was designated as a *Bacillus altitudinis* strain using 16S rDNA sequencing. The endophytic *B. altitudinis* had a notable influence on plant growth. The results of our assays revealed that the endophytic *B. altitudinis* raised the growth of different plant species. Remarkably, we found transcriptional changes in plants treated with the bacterium. Genes such as maturase K, tetratricopeptide repeat-like superfamily protein, LOB domain-containing protein, and BTB/POZ/TAZ domain-containing protein were highly expressed. In addition, we identified for the first time an induction in the endophytic bacterium of the major facilitator superfamily transporter and DNA gyrase subunit B genes during interaction with the plant. These new findings show that endophytic *B. altitudinis* could be used as a favourable candidate source to enhance plant growth in sustainable agriculture.

## Introduction

The use of fertilisers in agricultural practice to increase yields has had harmful effects on the environment and quality of the soil for a long time (Worlanyo and Jiangfeng, 2021). To minimise these negative impacts, the use of microorganisms located in the rhizosphere and in endophytic environments, such as bio-fertilisers, has received increased attention (Atieno et al., 2020). Beneficial plant–bacteria communities have been studied for many years (Pieterse et al., 2012; Zipfel and Oldroyd, 2017; Afzal et al., 2019; Ke et al., 2021; Song et al., 2021; Zhang et al., 2021). There are different bacteria in a direct interconnection with the plant cells; therefore, they might produce a direct beneficial effect (Hallmann et al., 1997). Different plant species are colonised by a great diversity of endophytic bacteria, such as *Pseudomonas*, *Bacillus*, *Enterobacter*, *Burkholderia*, and *Azospirillum* (Lodewyckx et al., 2002; Weyens et al., 2009).

The range of endophytic bacteria depends on the plant species and crop conditions (Zinniel et al., 2002; Ulrich et al., 2008). Endophytic bacteria colonise the intracellular and intercellular spaces of plant tissues and promote plant growth (Ulrich et al., 2008; Lugtenberg and Kamilova, 2009; Vacheron et al., 2013; Hardoim et al., 2015; Santoyo et al., 2016; Gouda et al., 2018). The procedures used by endophytic bacteria to boost plant growth constitute an essential step in the endophytic bacteria applications (Wani et al., 2015). However, the process by which endophytic bacteria enhance plant growth are not well understood (Hardoim et al., 2008; Marasco et al., 2012; Rashid et al., 2012; Bruto et al., 2014). Important evidence suggests that there is a relationship between genes involved in beneficial features (Bruto et al., 2014). The useful functions comprise the bacterial ability to enhance nutrient acquisition, fix nitrogen, acquire iron, and produce siderophores in plant species (Zhang et al., 2009; Hardoim et al., 2015; Zhou et al., 2016).

Furthermore, endophytic bacteria enhance plant growth through the production of different phytohormones. Phytohormones produced during endophytic colonisation process improved plant nutrient absorption and biomass (Gravel et al., 2007; Phetcharat and Duangpaeng, 2012; Shi et al., 2014). Plant growth regulators such as cytokines, ethylene, abscisic acid (ABA), gibberellins, and indole-3-acetic acid (IAA) are produced by endophytic bacteria during interactions with plants (Vacheron et al., 2013). Interestingly, IAA showed a crucial effect on plant growth and development processes. Modulating IAA is a notable hallmark through which the endophytic bacteria might strengthen plant growth. In addition, ethylene constitutes an essential plant hormone involved in different developmental and physiological processes (Sun et al., 2016). The enzyme 1-aminocyclopropane-1-carboxylate (ACC) deaminase precursor of ethylene is produced by endophytic bacteria (Sun et al., 2009). The hydrolysis of ACC improved plant growth under stress conditions (Santoyo et al., 2016). Plant growth-promoting endophytic bacteria displayed ACC deaminase activity (Nikolic et al., 2011; Zhang et al., 2011; Rashid et al., 2012). These bacteria can produce ACC deaminase to enhance plant growth. Mutation of the ACC deaminase gene compromised the canola growth (Sun et al., 2009). Moreover, diverse endophytic bacterial strains have the ability to release volatile organic compounds such as acetoin, pentadecane, 2,3-butanediol, 1 hexanol, and indole, which enhance plant growth (Ryu et al., 2003; Gutiérrez-Luna et al., 2010; Blom et al., 2011; Hernández-Calderón et al., 2018).

Plant endophytic bacteria have great potential to be used as bio-fertilisers. Although different types of bacterial species were identified from a broad plant species range, the action of these bacteria is commonly unstable under field conditions (Afzal et al., 2019). The poor understanding of the tricky events that control this alliance at the molecular level might be the reason. However, it is possible to obtain a better understanding through the evaluation of the both bacterial and plant genes expressed during the interaction (Afzal et al., 2019). The development of RNA-sequencing techniques has allowed the identification of transcripts involved in plant growth. However, few studies have investigated the endophytic bacterial diversity in unexplored wild plant species. Wild plant species survive in different environments and under diverse stress conditions. Identification of endophytic bacteria isolated from these species might allow the use of diverse mechanisms in these bacteria to promote plant growth (Afzal et al., 2019). The present study aimed to isolate, identify, and characterise endophytic bacteria from wild plant species and characterise their plant growth-promoting properties and the main molecular events involved in endophytic bacteria–plant interactions.

## Materials and Methods

### Isolation of Endophytic Bacterium

The samples were collected from wild plant species *Glyceria chinensis* (Keng) localised along the margin of the Fu Tuan River (35°20′17″N, 119°26′8″E) within 5 km^1^ of the coastal region of Rizhao City in Shandong Province (People’s Republic of China). The wild plant species was classified according to data on morphological traits from the Flora of China.^2^ This experimental study complies with Chinese national and local laws, and sample collection has been permitted by the Rizhao administration and Municipal Sciences and Technology Department [Collection information: South China Botanical Garden (IBSC) of the Chinese Academy of Sciences. Source: China Digital Plant Specimens Museum. Identifier: 0114164. Collector: Zhang Zhisong Acquisition number: 401467]. A total of 100 samples were collected randomly during the spring season. Firstly, the plant material (stems and roots) was rinsed with water. The samples were cut into small pieces under aseptic conditions. Each sample was surface sterilised with 70% ethanol for 1 min and immersed in a sodium hypochlorite solution (5%) during 1 min. Subsequently, the samples were washed in sterile distilled water during 1 min and dried on filter paper. After proper drying, pieces of plant parts were physically treated in a TissueLyser (Qiagen, Hilden, Germany) during 5 min in 1 ml of sterile water. The debris was decanted and 100 μl of the remaining water was incubated in Luria–Bertani (LB) agar medium (yeast extract, 5 g/L: peptone, 10 g/L; sodium chloride, 5 g/L; agar, 12 g/L; pH 7) at 37°C for 3 days. Parallel to the samples, the final wash solution of the surface sterilisation procedure was also spread plated onto the MS medium plate which served as a control. The bacteria were only isolated from processed samples internally. This was the criteria to classify as endophytes and not the surface contaminates. Bacterial colonies were selected based on growth rate, colony morphology, and pigmentation. Bacterial isolates were selected and purified by streaking procedure. These were incubated at 37°C. Pure cultures of the bacterial strains were maintained in 30% glycerol at −80°C.

### Plant Materials and Growth Conditions

*Arabidopsis thaliana* ecotype Columbia plants were used in the experiments. First, surface-sterilised seeds were plated on Murashige and Skoog (0.5 × MS) basal media (Sigma Aldrich, St. Louis, MO, United States) supplemented with 1% w/v sucrose. The seeds were stored at 4°C in the dark for 2 days and transferred to a controlled growth room under a 16-h light/8-h dark photoperiod at 22°C. Small plants were transplanted to a mixture of soil composed of peat plugs and vermiculite in a 1:1 ratio during 14 days. In addition, *Nicotiana tabacum*, corn, and soybean seeds were germinated and the plants grown in pots (6”) containing sterilised black turf and rice husk (4:1) substrate and maintained in a controlled growth room at 23°C.

### Identification of Endophytic Bacterium

The DNA from endophytic bacterium was extracted according to Sambrook et al. (1989). The 16S rRNA gene was used for molecular identification. A fragment of 16S rRNA gene was amplified by polymerase chain reaction (PCR) using the forward primer 27F 5′-AGAGTTTGATCCTGGCTCAG-3′ and reverse primer 1492R 5′-GGTTACCTTGTTACGACTT-3′. The amplification process was conducted in a Thermal Cycler T100 machine (Bio-Rad Life Science Research, Shanghai, People’s Republic of China) using a Taq PCR Master Mix Kit (Qiagen, Hilden, Germany). The PCR reaction was as follows: 95°C for 10 min; 35 cycles of 95°C for 30 s, 55°C for 1 min, and 72°C for 1.5 min; and final extension at 72°C for 10 min. The purified PCR fragment was sequenced using an ABI 3730 DNA sequencer (Applied Biosystems, ThermoFisher Scientific, Chino, CA, United States). The 16S rRNA gene fragment sequence (1,147 bp) was identified using BLASTN homology searches (Altschul et al., 1997). The National Center for Biotechnology Information (NCBI) GenBank, European Molecular Biology Laboratory–European Bioinformatics Institute (EMBL–EBI), and taxonomically united database of 16S rRNA EzBioCloud^2^ were used as databases (Yoon et al., 2017; De Los Santos Villalobos et al., 2019; Ha et al., 2019). Additionally, the full 16S rRNA gene (1,544 bp), glycosyl hydrolase lipoprotein, and SDR family oxidoreductase genes sequenced through RNA sequencing were used to an accurate identification. The phylogenetic tree was done using the neighbour-joining method and the full 16S rRNA (Saitou and Nei, 1987).

### Greenhouse Experiments

Seeds pre-germinated from *A. thaliana*, *N. tabacum*, corn, and soybean were treated with endophytic bacterial fermentation twice weekly for 1 month. The selected bacterial strain was incubated in 100 ml of LB broth medium in a 250-ml Erlenmeyer flask with shaking (200 rpm) for 1 day at 37°C in the dark. The optical density (OD) from endophytic bacterial fermentation was adjusted to 1.0 (4.77 × 10^9^ CFU/ml), and 30 ml of the fermentation was applied per pot. Each plastic pot received three pre-germinated seeds, and the plants were grown in a controlled growth room at 25°C and irrigated with water without any fertilisers. A completely randomised pot experiment with five replicates for each treatment was done to analyse the influence of the endophytic bacterium on the growing of different plant species. The substrate contained black turf and rice husks (4:1). The substrate was sterilised at 120°C during 20 min. Additionally, the roots of the plants inoculated with the endophytic bacterium used in the experiments were processed, and the bacterium was again isolated and classified as the original strain using the same protocol mentioned above. In *Arabidopsis* plants, the rosette diameter, leaf size, root size, and fresh weight parameters were evaluated after 1 month of treatment with endophytic bacterial fermentation. In addition, colony-forming units from endophytic bacteria were determined after 1 month of treatment. Plant size and fresh weight were evaluated in *N. tabacum*, corn, and soybean plants after 1 month of treatment. Data were analysed using GraphPad Prism software (La Jolla, CA, United States). Significant differences among the mean values were determined using *t*-test at *p* < 0.05. Five replicates were used for each treatment. The experiments were replicated twice.

### Identification of New Genes Using RNA Sequencing

The *Arabidopsis* plants were treated with aliquots of 30 ml (4.77 × 10^9^ CFU/ml) of endophytic bacterium. The bacterium was inoculated in the sterilised substrate around the plants (5-day-old). The colonisation of bacterium in the roots was verified using the same protocol used in the isolation of this strain from wild plant species on LB plates at different time points. The endophytic bacterium was well established 72 h post-inoculation. Leaves, stems, and roots from five plants were collected 72 h post-inoculation. Plants treated with water were used as control. Treatments and controls were repeated three times per group. The total RNA was extracted using the Qiagen RNeasy Midi Kit (Hilden, Germany), and the concentration of the total RNA was determined using spectrometry. The treatment and control samples were repeated three times per group. After extracting the total RNA, eukaryotic mRNA was enriched using oligo(dT) beads. The samples were sequenced using Illumina HiSeq^TM^ 2000 (Personalbio Co., Shanghai, People’s Republic of China). High-quality reads were processed using the Perl script, and the differentially expressed genes were identified using the edgeR package.^3^ Genes with a fold change ≥2 were considered significant differentially expressed genes. Gene ontology (GO) and the Kyoto Encyclopedia of Genes and Genomes (KEGG) pathway enrichment analysis were used to characterise the differentially expressed genes. GO functional annotations were obtained from the non-redundant annotation results. In addition, the GO annotations were analysed using the Blast2GO software^4^ (Conesa et al., 2005). The RNA sequencing was done following these steps: (1) total RNA extraction; (2) total RNA quality test; (3) rRNA removal; (4) RNA fragmentation between 200 and 300 bp by ion disruption; (5) PCR: the first chain of cDNA was synthesised by random primers and reverse transcriptase; when the second chain of cDNA was synthesised, the library of chain-specific components was constructed, and dTTP was replaced by dUTP, which greatly improved the accuracy of the results. (6) PCR enrichment of library fragments: the size of the library ranged from 300 to 400 bp. (7) Library quality inspection: the size of the library was detected by Agilent 2100 Bioanalyzer, and the total concentration of the library was detected by fluorescence quantitative analysis. (8) Illumina sequencing: the best amount of sample was selected for PCR amplification. (9) Annealing of sequencing at the same time of synthesis: the prokaryote genetic analysis process firstly filtered the raw off-machine data to obtain a high-quality sequence, comparing the filtered sequence with the species reference genome. Based on the results of the comparison, the amount of expression of each gene was calculated. On this basis, the expression difference analysis, enrichment analysis, and cluster analysis of the sample were further carried out. The transcription structure of the sample was analysed and the manipulative substructure of the sample gene obtained. UTR, SNP, and InDel were obtained. HTSeq 0.6.1p2^5^ was used to compare the read count values from each gene as the original expression of the gene. To make gene expression levels comparable between different genes and samples, we normalised the sequencing depth and gene length using fragments per kilobase of transcript per million (FPKM). FPKM calculates the number of fragments that two reads can compare to the same transcript. In the reference transcription group, we generally took into account the FPKM >1 when the gene was expressed. Pearson correlation coefficients were used to indicate the level of expression correlation between genes in the sample to test the reliability of the experiments and whether sample selection was reasonable. The level correlation of gene expression between samples was an important index to test the reliability of the experiments and whether sample selection was reasonable. The log2 fold change was calculated by dividing the FPKM values from the bacterium during interaction with *Arabidopsis* (SB001) and the bacterium without interaction with *Arabidopsis* (B001).

## Results

### Isolation and Identification of the Endophytic Bacterium *Bacillus altitudinis*

Six endophytic bacteria strains were isolated from wild plant species. The ability of the six isolated endophytic bacteria to colonise and persist in wild plant species was tested. Of these six, only one isolate (SB001) colonised this plant species at levels ranging from 9.40 × 10^2^ CFU/cm fresh root compared with the other five strains, where the level was lower (Table 1). Based on the levels of colonisation, the isolated endophytic strain SB001 was selected for further assays.

**TABLE 1:** Total bacterial population at harvest.

Samples	Root endosphere (10^2^ CFU/cm fresh root)^a^
SB001	9.40 ± 0.75
SB002	0.10 ± 0.01
SB003	2.50 ± 0.02
SB004	1.10 ± 0.04
SB005	0.20 ± 0.01
SB006	0.50 ± 0.02

*^*a*^Means of five replicates.*

**FIGURE 1:**
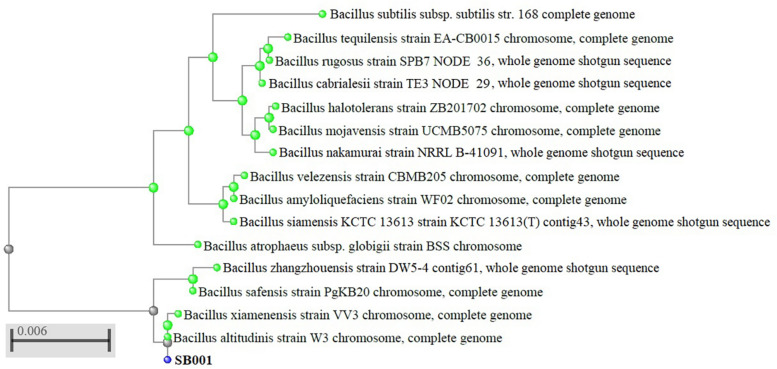
Phylogenetic tree of 16S rRNA sequences from endophytic bacterium strain (SB001) compared with representative members of *Bacillus* genus with more than 98% of identity.

The isolate SB001 showed a small rod-shaped structure and Gram-positive spore-forming bacterium distinctive of the genus *Bacillus*. The bacterium strain was identified using the partial (1,147 bp) and full (1,544 bp) 16S rRNA gene. Using the taxonomically united database of 16S rRNA in EzBioCloud, the full 16S rRNA was definitively identified as a top hit with *Bacillus altitudinis* with 100% similarity. Partial 16S rRNA sequences had an ambiguous 96.94% of similarity with *B. altitudinis*. Additionally, glycosyl hydrolase lipoprotein and SDR family oxidoreductase genes confirmed the identification of *B. altitudinis* (Supplementary Table 1). Considering the identification, the phylogenetic tree was constructed by comparing the full 16S rRNA gene sequences of the isolate SB001 with the reference strain sequences from the NCBI GenBank public database. Molecular analysis indicated that the isolated strain SB001 belongs to the genus *Bacillus* showing an identity percentage of 100% and *E*-value of 0.0 with *B. altitudinis* strain W3 (Figure 1).

### *Bacillus altitudinis* Strain Promotes the Growth of Different Plant Species

**FIGURE 2:**
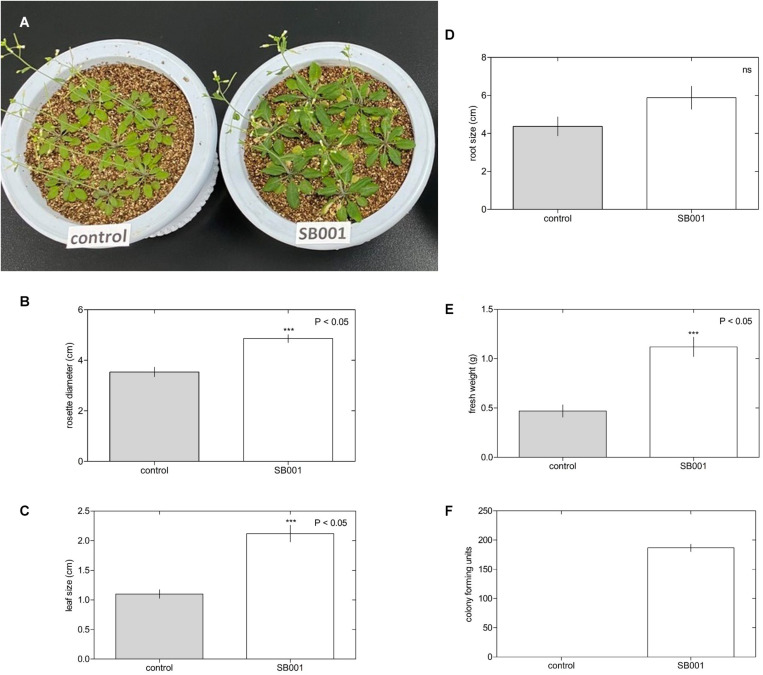
Endophytic bacterium *B. altitudinis* induces significant *Arabidopsis* plant growth. Plants (5-day-old) were mock-treated (control) or inoculated with 30 ml of *B. altitudinis* at 4.77 × 10^9^ CFU/ml. **(A)** Phenotype of *Arabidopsis* plants mock-treated (control) and treated with *B. altitudinis* fermentations (SB001) at 20 days post-inoculation. Effect on the rosette diameter **(B)**, leaf size **(C)**, root size **(D)**, and fresh weight **(E)** of *Arabidopsis* plants treated with *B. altitudinis* fermentations (SB001). **(F)** Colony-forming units were determined in *Arabidopsis* plants mock-treated (control) and treated with *B. altitudinis* fermentations (SB001). Each bar represents mean values with standard errors of two independent experiments (*n* = 10). ***P ≤ 0.001.

The endophytic strain was also evaluated for plant growth promotion in different plant species. There was significant growth in *Arabidopsis* plants treated with the endophytic bacterium *B. altitudinis* (Figure 2A). The rosette diameter (Figure 2B), leaf size (Figure 2C), and fresh weight (Figure 2E) were considerably increased by inoculation with the *B. altitudinis* strain compared with the control in *Arabidopsis* plants at 20 days. However, endophytic bacteria did not influence root size (Figure 2D). Nevertheless, endophytic bacteria were suitably established in *Arabidopsis* roots (Figure 2F).

**FIGURE 3:**
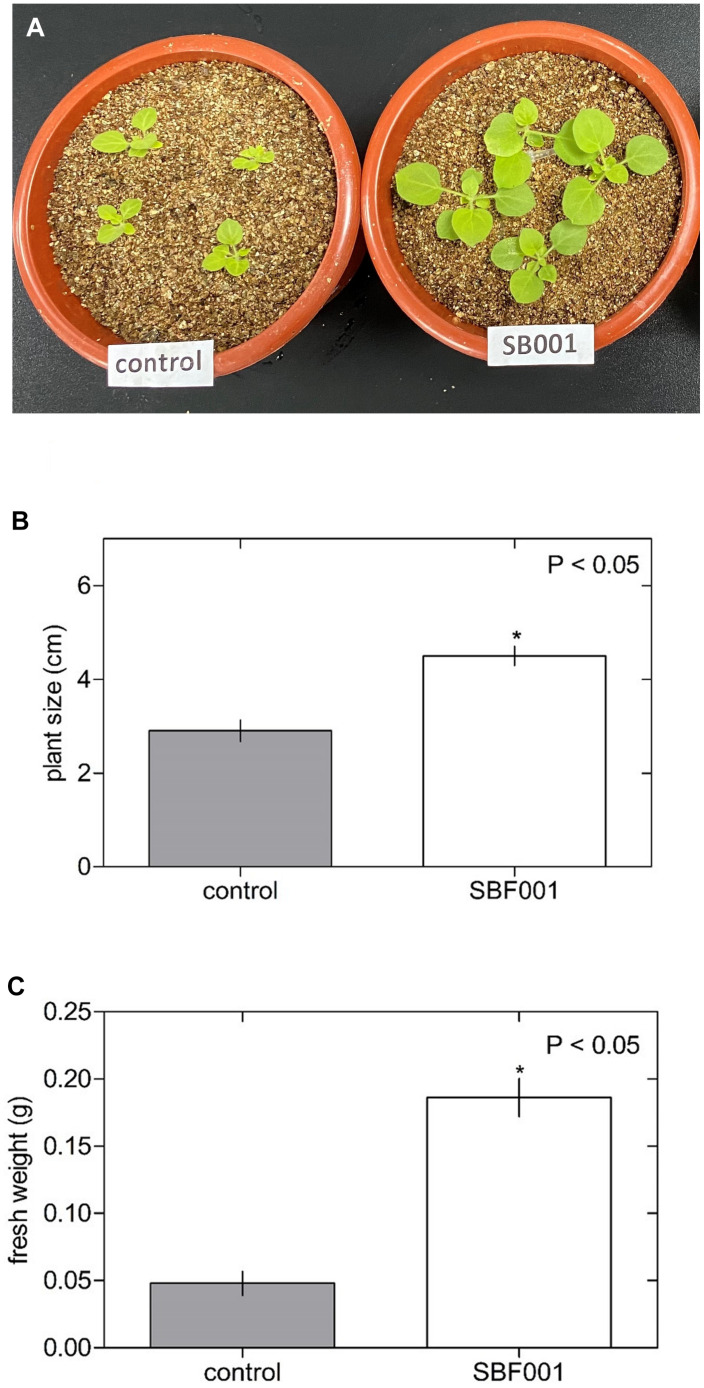
Endophytic bacterium *B. altitudinis* induces significant *Nicotiana tabacum* plant growth. Plants (5-day-old) were mock-treated (control) or inoculated with 30 ml of *B. altitudinis* at 4.77 × 10^9^ CFU/ml. **(A)** Phenotype of *N. tabacum* plants mock-treated (control) and treated with *B. altitudinis* fermentations (SB001) at 20 days post-inoculation. Effect on plant size **(B)** and fresh weight **(C)** of *N. tabacum* plants mock-treated (control) and treated with *B. altitudinis* fermentations (SB001). Each bar represents mean values with standard errors of two independent experiments (*n* = 10). *P ≤ 0.05.

**FIGURE 4:**
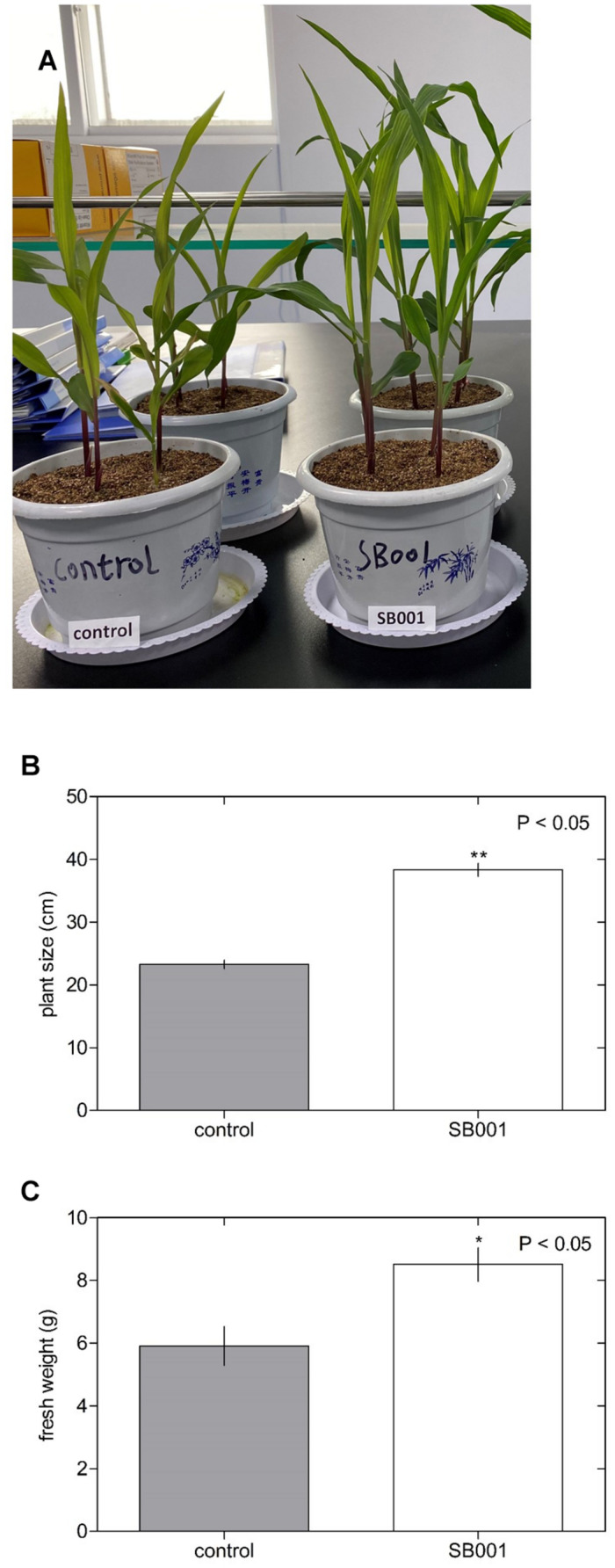
Endophytic bacterium *B. altitudinis* induces significant maize plant growth. Plants (5-day-old) were mock-treated (control) or inoculated with 30 ml of *B. altitudinis* at 4.77 × 10^9^ CFU/ml. **(A)** Phenotype of maize plants mock-treated (control) and treated with *B. altitudinis* fermentations (SB001) at 20 days post-inoculation. Effect on plant size **(B)** and fresh weight **(C)** of maize plants mock-treated (control) and treated with *B. altitudinis* fermentations (SB001). Each bar represents mean values with standard errors of two independent experiments (*n* = 10). *P ≤ 0.05; **P ≤ 0.01.

**FIGURE 5:**
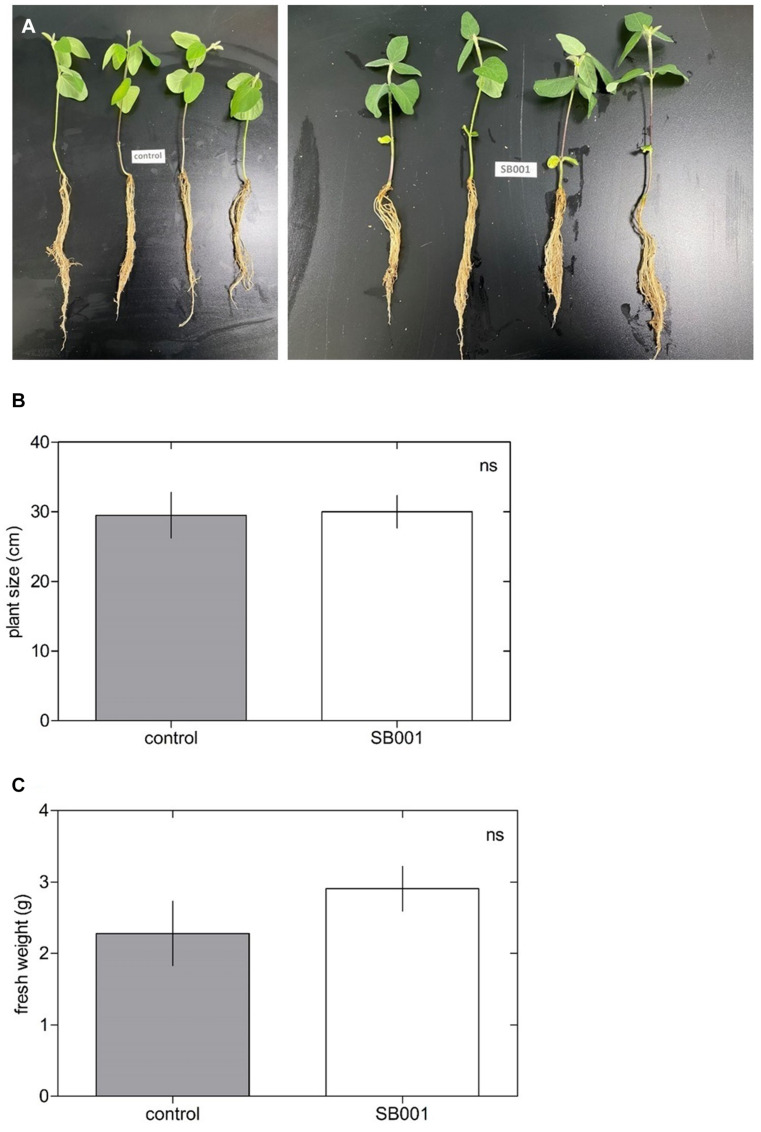
Endophytic bacterium *B. altitudinis* does not induce soybean plant growth. Plants (5-day-old) were mock-treated (control) or inoculated with 30 ml of *B. altitudinis* at 4.77 × 10^9^ CFU/ml. **(A)** Phenotype of soybean plants mock-treated (control) and treated with *B. altitudinis* fermentations (SB001) at 20 days post-inoculation. Effect on plant size **(B)** and fresh weight **(C)** of soybean plants mock-treated (control) and treated with *B. altitudinis* fermentations (SB001). Each bar represents mean values with standard errors of two independent experiments (*n* = 10).

The *B. altitudinis* strain significantly stimulated the growth of *N. tabacum* plants (Figure 3A) and showed a substantial influence on plant size (Figure 3B) and fresh weight (Figure 3C) in *N. tabacum* plants. Inoculation with the *B. altitudinis* strain considerably enhanced the growth of maize plants with respect to non-inoculated plants (Figure 4A). Compared with the non-inoculated plants, the endophytic strain increased the plant size (Figure 4B) and fresh weight (Figure 4C) in inoculated plants. However, the endophytic strain had no significant influence on soybean plant growth (Figure 5). Overall, these results revealed that *B. altitudinis* improved the vegetative growth of different plant species following inoculation.

### *Bacillus altitudinis* Induces Transcriptional Changes in Genes Involved in Plant Growth

**FIGURE 6:**
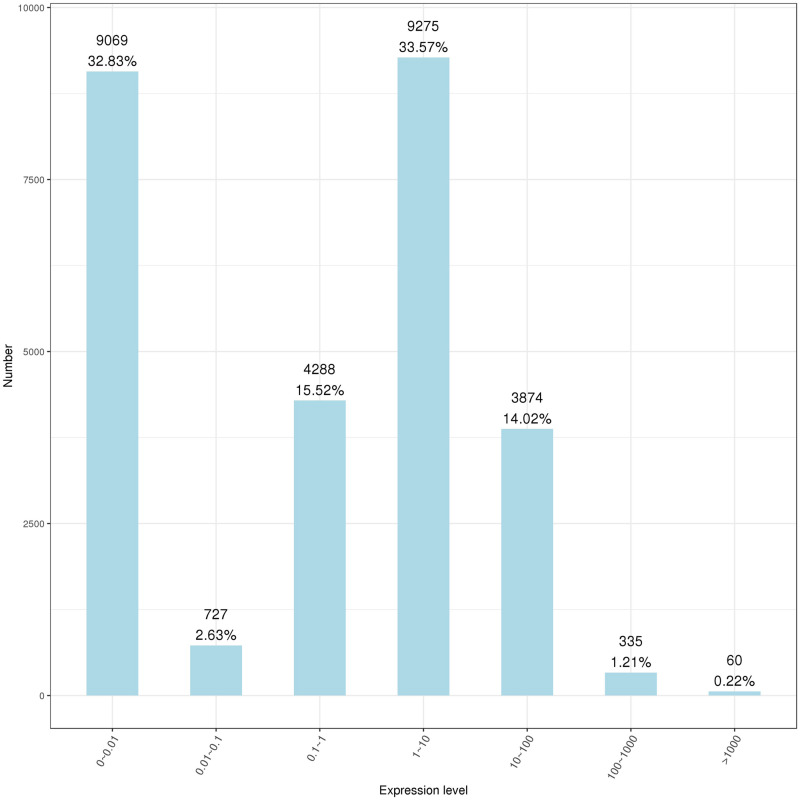
Statistics showing the range of plant gene expressions during the *B. altitudinis*–*Arabidopsis* interaction. Horizontal coordinates represent the range of expression measures, and ordinates represent the number of genes in each expression interval.

**FIGURE 7:**
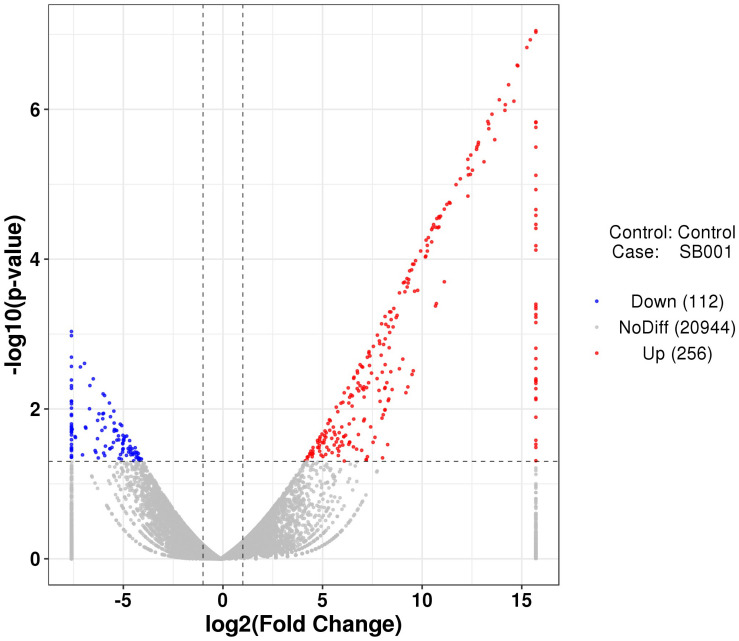
Volcanic diagrams of differential expression genes. The horizontal coordinates are log2 (fold change) and the ordinates are -log10 (*p*-value). The two vertical dashed lines are 2 times the difference threshold, and the horizontal dashed lines are *p*-value 0.05 thresholds. A red dot indicates the upregulated genes group, a blue dot indicates the downregulated genes group, and a grey dot represents a non-significant difference in the expression of the genes group. The scale was fixed considering the minimum and maximum log2 values (between -7.60 and 15.70) included in the Supplementary Table 4.

RNA-seq was used to evaluate the interactions between *B. altitudinis* and *Arabidopsis*. To generate RNA-seq data sets, we prepared cDNA libraries from mock-inoculated and inoculated with the endophytic bacterium *B. altitudinis* from *Arabidopsis* plant RNA and sequenced them on two separate flow cells. The range of gene expression during the *B. altitudinis*–*Arabidopsis* interaction was determined. During the interaction, 27,628 transcripts were identified and annotated (Supplementary Table 2). From this, 7,379 new transcripts were annotated (Supplementary Table 3). The highest number of transcripts (33.57%) showed an expression level change of between 1- and 10-fold. The lowest number of transcripts was distributed at the highest expression level between >100-fold change (Figure 6). Analysis of RNA-seq data during the interaction revealed 368 differentially expressed transcripts, which included 256 upregulated and 112 downregulated transcripts (Figure 7). Among them, maturase K, tetratricopeptide repeat (TPR)-like superfamily protein, LOB domain-containing protein, and BTB/POZ and TAZ domain-containing proteins showed the highest gene differential expression during *B. altitudinis*–*Arabidopsis* interaction. Trihelix transcription factor (TF) ASR3, TF MYB90, and *O*-methyltransferase family proteins were significantly repressed during the interaction (Table 2 and Supplementary Table 4).

**TABLE 2:** Most important differentially expressed genes in *Arabidopsis* plants inoculated with *B. altitudinis*.

ID	Log2 fold change	Description
**Upregulated genes**		
ATCG00040	12.8	Maturase K
ATCG00360	12.3	Tetratricopeptide repeat (TPR)-like superfamily protein
AT4G37540	11.1	LOB domain-containing protein
AT3G48360	10.7	BTB/POZ and TAZ domain-containing protein
AT5G50915	9.5	Transcription factor bHLH137
AT4G28040	9.2	WAT1-related protein
AT5G09730	9.1	Beta-D-xylosidase
AT4G37390	8.9	Auxin-responsive GH3 family protein
AT4G16590	8.7	Cellulose synthase-like A01
AT4G15210	8.4	Beta-amylase
AT3G45140	8.1	Lipoxygenase 2
AT5G44050	8.1	Protein DETOXIFICATION 28
AT1G54020	8.0	GDSL esterase/lipase At1g54020
AT1G52400	8.0	Beta-D-glucopyranosyl abscisate beta-glucosidase
AT3G47340	7.9	DIN6
AT2G39030	7.9	*L*-ornithine N5-acetyltransferase NATA1
AT1G01480	7.8	1-Aminocyclopropane-1-carboxylate synthase
AT5G20630	7.5	Germin-like protein subfamily
AT4G36850	7.4	PQ-loop repeat family protein
AT4G22755	7.3	SMO1–3
AT3G44300	7.2	NIT2
AT1G73260	7.2	Kunitz trypsin inhibitor 1
AT4G21680	7.1	Protein NRT1/PTR family
AT1G02205	7.1	Fatty acid hydroxylase superfamily
ATMG00570	7.0	Sec-independent periplasmic protein translocase
ATCG00190	6.6	DNA-directed RNA polymerase subunit beta
AT2G43620	6.6	Endochitinase At2g43620
AT4G37150	6.1	MES9
AT2G25900	6.0	Zinc finger CCCH domain-containing protein
AT3G16240	5.9	Aquaporin TIP2-1
AT5G12940	5.9	Leucine-rich repeat (LRR) family protein
AT5G03260	5.8	Laccase
AT4G23600	5.7	Cystine lyase CORI3
AT1G09350	5.6	Hexosyltransferase
AT1G76930	5.6	Extensin
AT1G54010	5.5	Inactive GDSL esterase/lipase-like protein
AT1G03220	5.4	Eukaryotic aspartyl protease family protein
AT1G52040	5.4	Myrosinase-binding protein
AT2G39800	5.3	Delta-1-pyrroline-5-carboxylate synthase A
AT4G01870	5.1	TolB protein-related
AT2G05790	5.1	*O*-glycosyl hydrolases family 17 protein
AT2G07715	5.1	Nucleic acid-binding, OB-fold-like protein
AT1G05680	5.0	Glycosyltransferase
AT2G23130	5.0	Lysine-rich arabinogalactan protein
AT5G20250	5.0	Raffinose synthase family protein
AT5G40890	4.9	Chloride channel protein CLC-a
AT2G06850	4.9	Xyloglucan endotransglucosylase/hydrolase protein
AT1G78850	4.9	EP1-like glycoprotein
AT2G29350	4.8	SAG13
AT4G12420	4.8	Monocopper oxidase-like protein SKU5
AT5G25980	4.7	Myrosinase
AT3G01500	4.5	Beta carbonic anhydrase 1, chloroplastic
AT1G44350	4.5	IAA-amino acid hydrolase
AT4G34710	4.4	Arginine decarboxylase
AT1G77760	4.3	Nitrate reductase
AT1G45201	4.1	Triacylglycerol lipase-like 1
**Downregulated genes**		
AT2G33550	−7.4	Trihelix transcription factor ASR3
AT1G66390	−7.1	Transcription factor MYB90
AT1G21120	−7.0	*O*-methyltransferase family protein
AT2G28720	−6.3	Histone
AT1G56250	−6.2	F-box protein VBF
AT2G16600	−6.0	Peptidyl-prolyl cis–trans isomerase
AT5G23750	−5.6	Remorin family protein
AT3G52280	−5.5	General transcription factor group E6
AT1G68790	−5.4	Protein CROWDED NUCLEI
AT2G40880	−5.3	Cysteine proteinase inhibitor
AT3G51920	−5.1	Calmodulin-like protein
AT5G05410	−5.1	Dehydration-responsive element-binding protein 2A
AT5G52740	−5.1	Heavy metal-associated isoprenylated plant protein 12
AT3G51910	−5.0	Heat stress transcription factor A-7a
AT5G16570	−5.0	Glutamine synthetase
AT5G16470	−5.0	Protein METHYLENE BLUE SENSITIVITY 2
AT1G76720	−4.9	Eukaryotic translation initiation factor 2 (eIF-2)
AT5G40340	−4.9	Tudor/PWWP/MBT superfamily protein
AT1G22160	−4.9	FCS-like zinc finger 5
AT5G39950	−4.8	Thioredoxin H2
AT5G55660	−4.3	DEK domain-containing chromatin associated protein
AT5G52640	−4.3	Heat shock protein 90
AT4G19840	−4.3	Protein PHLOEM PROTEIN 2-LIKE A1
AT4G29160	−4.2	Vacuolar protein sorting-associated protein
AT3G25230	−4.2	Peptidylprolyl isomerase
AT3G52400	−4.0	Syntaxin
AT2G33550	−7.4	Trihelix transcription factor ASR3

**FIGURE 8:**
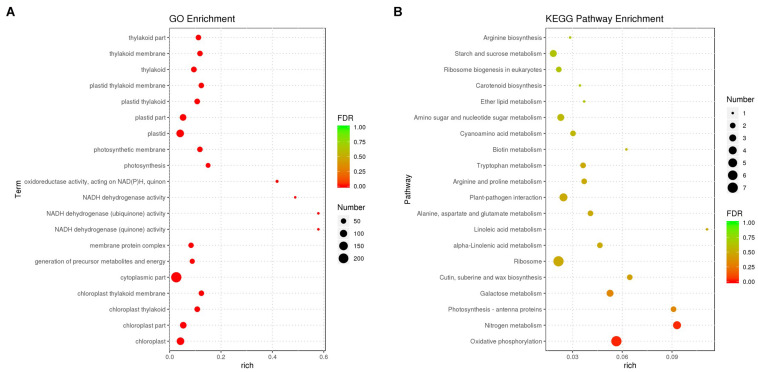
Gene ontology (GO) **(A)** and KEGG pathway-rich analysis **(B)**. Based on the GO-rich and KEGG results, the extent of richness is measured by the rich factor, false discovery rate (FDR) values, and the number of genes that were collected into this GO term or KEGG pathway. Rich factor refers to the ratio of the number of different genes collected in the GO term or KEGG pathway to the number of genes annotated.

**FIGURE 9:**
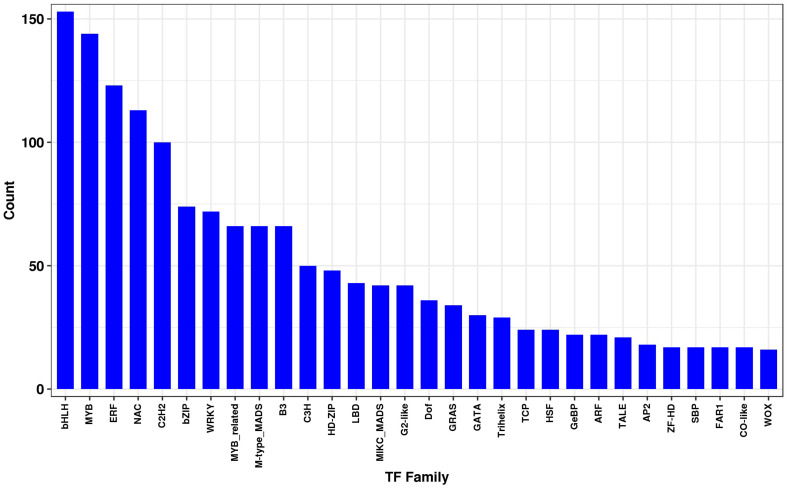
Transcription factor family. The horizontal coordinates are different transcription factor families, and the ordinates are the number of genes that fall into that transcription factor family.

Differentially expressed transcripts were subjected to functional analyses. GO enrichment analysis was performed using the annotated difference gene to calculate the list of genes and the number of genes for each term. The transcripts with the largest increases in expression were NADH dehydrogenase (quinone), NADH dehydrogenase (ubiquinone) activity, NADH dehydrogenase, and oxidoreductase (Figure 8A). Based on the KEGG pathway, we categorised the most significant transcripts into nitrogen metabolism and oxidative phosphorylation pathways (Figure 8B and Supplementary Table 5). The expression profile of TF genes displays the upcoming transcription actions regulated by these genes. To determine their important roles, the TFs involved in the *B. altitudinis*–*Arabidopsis* interaction were analysed. The largest TF families detected in our study were basic helix-loop-helix (bHLH) DNA-binding superfamily protein, TF MYB13 (MYB), ethylene responsive factor (ERF), NAC domain-containing protein 1 (NAC), and zinc finger protein ZAT1 (C2H2), respectively (Figure 9).

During the interaction, a total of 3,413 genes were annotated from *B. altitudinis*. From these, genes with a log2 fold change between −1.2 and 5.3 were analysed. Twenty-two and 8 *B. altitudinis* genes were up- and down-regulated during the interaction with *Arabidopsis* plants, respectively. The rest of these genes were undefined, repeated, low significant fold change, or involved in the primary metabolism of the bacterium. The major facilitator superfamily (MFS) transporter, sucrose-6-phosphate hydrolase, phosphate ABC transporter, DNA gyrase subunit B, polysaccharide biosynthesis protein, serine phosphatase, and flagellar biosynthesis protein had the highest expression levels in *B. altitudinis* during the interaction with *Arabidopsis* plants. Moreover, elongation factors G, 2,3-butanediol dehydrogenase, dihydrolipoamide succinyltransferase, and proline dehydrogenase were highly downregulated in bacteria (Table 3 and Supplementary Table 6).

**TABLE 3:** Most important differentially expressed genes in *B. altitudinis* during the interaction with *Arabidopsis* plants.

ID	Log2 fold change	Swiss-Prot
**Upregulated genes**		
Gene2967	5.3	MFS transporter
Gene2185	4.5	Sucrose-6-phosphate hydrolase
Gene1412	4.0	Phosphate ABC transporter
Gene3573	3.5	DNA gyrase subunit B
Gene1888	3.3	Polysaccharide biosynthesis protein
Gene3614	3.3	Serine phosphatase
Gene273	3.3	Flagellar biosynthesis protein
Gene1747	2.7	Electron transfer flavoprotein subunit beta
Gene3085	2.5	Phosphoribosylaminoimidazole synthetase
Gene1805	2.3	Bifunctional oligoribonuclease/PAP phosphatase NrnA
Gene3086	2.3	Phosphoribosylglycinamide formyltransferase
Gene1799	2.2	Acetyl-carboxylase subunit beta
Gene1627	2.0	Rod shape-determining protein
Gene1555	1.7	Uridine kinase
Gene388	1.6	Cell division protein
Gene459	1.5	Branched-chain alpha-keto acid dehydrogenase subunit E2
Gene2681	1.4	3-Hydroxyacyl-CoA dehydrogenase
Gene1634	1.2	Valyl-tRNA synthetase
Gene2803	1.1	Alpha-mannosidase
Gene369	1.0	Isoleucine
Gene284	1.0	Flagellar basal body rod protein
Gene389	1.0	Cell division protein
**Downregulated genes**		
Gene3695	−3.8	Elongation factor G
Gene1531	−2.7	2,3-Butanediol dehydrogenase
Gene1048	−2.6	Dihydrolipoamide succinyltransferase succinyl transferase
Gene2911	−2.6	Proline dehydrogenase
Gene3548	−1.9	Single-stranded DNA-binding protein
Gene668	−1.7	Flagellin
Gene3183	−1.4	Crp/Fnr family transcriptional regulator
Gene3475	−1.2	Flotillin

## Discussion

Different classes of microorganism have the potential to promote plant growth. The favourable effects of endophytic bacteria derived from the roots have been demonstrated. The significant reaction of these endophytic bacteria on plant growth has been achieved by both direct and indirect processes. Direct effects comprise the production of molecules that encourage plant growth (Goswami et al., 2016). In the current study, different endophytic bacteria strains were isolated from wild plant species. The SB001 strain was the most abundant in the inner tissues of the wild plant species. The strain was identified as *B. altitudinis* through morphological and molecular analyses.

Several plant growth promotion assays were conducted to determine whether SB001 could promote plant growth in different plant species under greenhouse conditions. Inoculated *A. thaliana*, *N. tabacum*, and maize plants exhibited growth improvement, and significant increases were observed for several growth variables, such as fresh weight and plant size, between the inoculated and non-inoculated control plants. However, this strain did not influence soybean growth. It is likely that the internal tissue environment of soybean roots is not suitable for the correct setting of the bacterium. Interestingly, *B. altitudinis* strain was well established in *Arabidopsis* roots, which constitutes important evidence of the ability of this strain to colonise internal tissues. Remarkably, this endophytic strain displayed vigorous plant growth-promoting effects in non-host plants. This result proposes that SB001 has the ability to efficiently enhance the growth of a broad range of plant species, and consequently, this might be used in applications under field conditions.

Earlier studies showed that several *Bacillus* species isolated from various hosts promote plant growth (Ashraf et al., 2004; Xie et al., 2014; Kuan et al., 2016; Pourbabaee et al., 2016; Radhakrishnan and Lee, 2016; Fouda et al., 2021). For example, *B. insolitus*, *B. subtilis*, and *B. methylotrophicus* increased the size and weight of shoots, leaves, and roots from diverse plant species (Ashraf et al., 2004; Radhakrishnan and Lee, 2016). Furthermore, *B. subtilis* and *B. methylotrophicus* induced the production of hormones such as IAA, cytokinins, gibberellic acid (GA), and spermidines to stimulate plant growth (Xie et al., 2014; Radhakrishnan and Lee, 2016; Fouda et al., 2021), whereas *B. megaterium* and *B. methylotrophicus* boosted endogenous proteins, amino acids, and minerals in plants (Kang et al., 2014; Radhakrishnan and Lee, 2016). Strain SB001 exhibited different plant growth-promoting properties, which were reflected in the enhanced vegetative growth of different plant species, although these properties under soil conditions need to be investigated. Due to its beneficial plant growth-promoting activities, the *B. altitudinis* strain may be used as a source of bio-fertiliser in sustainable agriculture.

Plant–harmless bacteria interactions have been studied for many years. However, the molecular events used and involved during the interaction are not well understood, which makes it difficult to use the advantages of these complex interactions under natural conditions. Endophytic bacteria positively impact plant growth and select beneficial bacterial colonisers (Hardoim et al., 2008; Marasco et al., 2012; Rashid et al., 2012). In this study, we conducted transcriptome sequencing of *Arabidopsis*–*B. altitudinis* interactions to identify and quantify the expression grades of transcripts. A large number of novel candidate genes related with primary and secondary metabolite biosynthesis were identified in our expressed sequence tag (EST) data set. The results show new understandings in the expression data of this endophytic bacterium, as well as a first insight into the gene expression patterns during *Arabidopsis*–*B. altitudinis* interaction. Moreover, the transcriptome information obtained in this study provides a significant contribution to gaining a suitable understanding of the molecular events implicated in the interaction.

Remarkably, GO and KEGG analyses showed significant changes, suggesting that the *B. altitudinis* strain had a considerable effect on the plant. Our data indicated that SB001 induces important pathways involved in plant growth. All the information about the molecular interactions in known metabolic and regulatory pathways are contained in the KEGG pathway database (Kanehisa and Goto, 2000). Interestingly, the maturase K (chloroplast-encoded splicing factor), TPR-like superfamily protein, LOB domain-containing protein, and BTB/POZ and TAZ domain-containing protein genes were highly induced for the first time during the *Arabidopsis*–*B. altitudinis* interaction.

Previously, maturase K gene expression was associated with developmental stage, etiolation, and regulation processes related with development and photosynthesis (Barthet and Hilu, 2007). Maturase K has influence in gene expression in plants and interferes with the proper expression of the photosynthetic mechanism (Qu et al., 2018). Indeed, the induction of the maturase K gene might have an effect on plant growth during the interaction, through an enhancing photosynthetic process. TPR proteins are important in plant hormone signalling, such as GA, cytokinin, and ethylene (Jacobsen et al., 1996; Wang et al., 2004; Greenboim-Wainberg et al., 2005; Yoshida et al., 2005). In addition, the TPR gene was a key positive regulator of ABA during *Arabidopsis* seedling development (Rosado et al., 2006).

Conversely, LOB domain-containing protein genes constitute a gene family that encodes plant-specific TFs that play key functions during plant growth (Zhang et al., 2020). These proteins are essential TFs that regulate plant organ development, photomorphogenesis (Mangeon et al., 2011), petiole development (Ge et al., 2014), hormone response (Zentella et al., 2007), and metabolism regulation (Rubin et al., 2009). Additionally, BTB/POZ and TAZ domain-containing protein genes are required during gametophyte development (Robert et al., 2009). This is an essential element of a signalling network that recognises and responds to diverse stimuli (Mandadi et al., 2009). Furthermore, the TF MYB90 is repressed during the interaction. Previously, it was found that heterologous expression of the TF AtMYB90 increased the anthocyanin production and inhibited the tomato plant growth (Li et al., 2018). The endophytic bacterium SB001 was capable of promoting growth through activation of important pathways implicated in plant growth, including the synthesis of phytohormones and TFs.

The TFs constitute crucial functional factors involved in the regulation of gene expression (Jiao et al., 2003). Numerous processes such as brassinosteroid signalling, ABA signalling, and axillary meristem formation are regulated by bHLH family members during seedling development (Zhao et al., 2012). A total of 153 transcripts were identified as bHLH TF proteins, and these constituted the largest TF family during the interaction. In addition, the bHLH proteins are a phytochrome interacting factor (Toledo-Ortiz et al., 2003).

The MYB proteins were involved in regulatory networks controlling development and metabolism of a diversity of plant species (Dubos et al., 2010). MYB115 and MYB118 had a key part in embryogenesis in *Arabidopsis* plants (Wang et al., 2009). Also, MYB38 and MYB18 regulate hypocotyl elongation in response to blue (Hong et al., 2008) and far-red light, respectively (Yang et al., 2009). Other MYB proteins, such as MYB58, MYB63, MYB85, MYB68, and MYB46, were related in the regulation of cell wall biosynthesis (Zhou et al., 2009). In this study, 144 putative MYB genes were detected during *Arabidopsis* seedling development.

The large NAC TF family was involved in a diversity of developmental events in *Arabidopsis* and soybean (Shamimuzzaman and Vodkin, 2013). This NAC TF family has the capacity to permit crosstalk between different pathways (Olsen et al., 2005). Furthermore, C2H2 zinc finger protein genes play a significant part in the development and differentiation (Razin et al., 2012). During the interaction, 100 putative C2H2 zinc finger genes were identified. In addition, ethylene responsive factor (ERF) TFs belong to the Apetala2/ERF) TF family involved in plant growth.

Interestingly, the MFS transporter and DNA gyrase subunit B protein genes were highly expressed in *B. altitudinis* during the interaction with the plant, which constitutes the first evidence of the expression of these types of genes in this species. The MFS transporter facilitates the transport of diverse molecules such as sugars, vitamins, amino acids, and hormones across cell membranes. These proteins are in different prokaryote and eukaryote organisms (Pao et al., 1998). It is likely that the high expression of this gene allows the transport of important hormones involved in plant growth. Silencing of the *A. thaliana* gyrase-A gene was embryo-lethal, whereas silencing of gyrase-B genes led to severely affected growth and development (Wall et al., 2004). We speculate that the overexpression of the gyrase gene in *B. altitudinis* might enhance plant growth.

## Conclusion

Here, we report a new *B. altitudinis* endophytic strain isolated from wild plant species. Our finding revealed that the endophytic bacterium induced a key group of genes associated with plant growth-promoting traits. We demonstrated that this strain has a favourable reaction on plant growth. Remarkably, our results suggest that maturase K, TPR-like superfamily protein, LOB domain-containing protein, and BTB/POZ/TAZ domain-containing protein might be involved for the first time in this process. Furthermore, the MFS transporter and DNA gyrase subunit B genes expressed in *B. altitudinis* may be implicated in the observed effects. Future studies should evaluate the direct functions of these genes in plant growth during interactions. In addition, the potential beneficial effects of *B. altitudinis* strain under natural conditions need to be further investigated. In the long term, the new finding would allow the development of suitable and effective strategies in sustainable agriculture.

## Data Availability Statement

The data presented in the study are deposited in the (SRX11022601) repository, accession number (PRJNA733570).

## Author Contributions

OB-H, HX, and XG conceived the study. OB-H and DZ designed the experiments. DZ, HX, JG, RP, LD, XG, CB, and OB-H did the experiments. DZ and OB-H assisted in the data analysis. OB-H wrote the manuscript. All authors contributed to the article and approved the submitted version.

## Conflict of Interest

DZ, HX, JG, RP, LD, XG, and OB-H were employed by the company Yota Bio-Engineering Co., Ltd. CB was employed by the company VBS Biotec SA, Mexico. The remaining author declare that the research was conducted in the absence of any commercial or financial relationships that could be construed as a potential conflict of interest.

## Footnotes

1 https://www.ezbiocloud.net

2 http://www.iplant.cn/foc/

3 www.r-project.org/

4 www.blast2go.com

5 http://htseq.readthedocs.io

## Supplementary Material

Supplementary Table 1 Identification of endophytic bacterium.

Supplementary Table 2 Annotation *Arabidopsis*–*B. altitudinis* interaction.

Supplementary Table 3 New transcript annotation *Arabidopsis*-SB001 interaction.

Supplementary Table 4 *Arabidopsis* vs. *B. altitudinis* up and down regulated genes during the interaction.

Supplementary Table 5 KEGG enrichment *Arabidopsis*–*B. altitudinis* interaction.

Supplementary Table 6 *B. altitudinis* bacterium.
