# Supplementary material for: Endophytic Bacillus altitudinis Strain Uses Different Novelty Molecular Pathways to Enhance Plant Growth
Source: Front Microbiol. 2021 Jun 25;12:692313. doi: 10.3389/fmicb.2021.692313 (PMC8268155; doi:10.3389/fmicb.2021.692313)
Supplement: Supplementary Table 1 — Identification of endophytic bacterium. [file Table_1.DOCX]

**TABLE 1** Total bacterial population at harvest.

| **Samples** | **Root-endosphere (10^2^ cfu cm^−1^ fresh root)**^a^ |
| --- | --- |
| SB001 | 9.40 ± 0.75 |
| SB002 | 0.10 ± 0.01 |
| SB003 | 2.50 ± 0.02 |
| SB004 | 1.10 ± 0.04 |
| SB005 | 0.20 ± 0.01 |
| SB006 | 0.50 ± 0.02 |

^a^ Means of five replicates

**TABLE 2** Most important differentially expressed genes in Arabidopsis plants inoculated with *B.* *altitudinis*

| **ID** | **Log2 Fold Change** | **Description** |
| --- | --- | --- |
| **Upregulated genes** | | |
| ATCG00040 | 12.8 | Maturase K |
| ATCG00360 | 12.3 | Tetratricopeptide repeat (TPR)-like superfamily protein |
| AT4G37540 | 11.1 | LOB domain-containing protein |
| AT3G48360 | 10.7 | BTB/POZ and TAZ domain-containing protein |
| AT5G50915 | 9.5 | Transcription factor bHLH137 |
| AT4G28040 | 9.2 | WAT1-related protein |
| AT5G09730 | 9.1 | Beta-D-xylosidase |
| AT4G37390 | 8.9 | Auxin-responsive GH3 family protein |
| AT4G16590 | 8.7 | Cellulose synthase-like A01 |
| AT4G15210 | 8.4 | Beta-amylase |
| AT3G45140 | 8.1 | Lipoxygenase 2 |
| AT5G44050 | 8.1 | Protein DETOXIFICATION 28 |
| AT1G54020 | 8.0 | GDSL esterase/lipase At1g54020 |
| AT1G52400 | 8.0 | Beta-D-glucopyranosyl abscisate beta-glucosidase |
| AT3G47340 | 7.9 | DIN6 |
| AT2G39030 | 7.9 | L-ornithine N5-acetyltransferase NATA1 |
| AT1G01480 | 7.8 | 1-aminocyclopropane-1-carboxylate synthase |
| AT5G20630 | 7.5 | Germin-like protein subfamily |
| AT4G36850 | 7.4 | PQ-loop repeat family protein |
| AT4G22755 | 7.3 | SMO1-3 |
| AT3G44300 | 7.2 | NIT2 |
| AT1G73260 | 7.2 | Kunitz trypsin inhibitor 1 |
| AT4G21680 | 7.1 | Protein NRT1/PTR family |
| AT1G02205 | 7.1 | Fatty acid hydroxylase superfamily |
| ATMG00570 | 7.0 | Sec-independent periplasmic protein translocase |
| ATCG00190 | 6.6 | DNA-directed RNA polymerase subunit beta |
| AT2G43620 | 6.6 | Endochitinase At2g43620 |
| AT4G37150 | 6.1 | MES9 |
| AT2G25900 | 6.0 | Zinc finger CCCH domain-containing protein |
| AT3G16240 | 5.9 | Aquaporin TIP2-1 |
| AT5G12940 | 5.9 | Leucine-rich repeat (LRR) family protein |
| AT5G03260 | 5.8 | Laccase |
| AT4G23600 | 5.7 | Cystine lyase CORI3 |
| AT1G09350 | 5.6 | Hexosyltransferase |
| AT1G76930 | 5.6 | Extensin |
| AT1G54010 | 5.5 | Inactive GDSL esterase/lipase-like protein |
| AT1G03220 | 5.4 | Eukaryotic aspartyl protease family protein |
| AT1G52040 | 5.4 | Myrosinase-binding protein |
| AT2G39800 | 5.3 | Delta-1-pyrroline-5-carboxylate synthase A |
| AT4G01870 | 5.1 | TolB protein-related |
| AT2G05790 | 5.1 | O-Glycosyl hydrolases family 17 protein |
| AT2G07715 | 5.1 | Nucleic acid-binding, OB-fold-like protein |
| AT1G05680 | 5.0 | Glycosyltransferase |
| AT2G23130 | 5.0 | Lysine-rich arabinogalactan protein |
| AT5G20250 | 5.0 | Raffinose synthase family protein |
| AT5G40890 | 4.9 | Chloride channel protein CLC-a |
| AT2G06850 | 4.9 | Xyloglucan endotransglucosylase/hydrolase protein |
| AT1G78850 | 4.9 | EP1-like glycoprotein |
| AT2G29350 | 4.8 | SAG13 |
| AT4G12420 | 4.8 | Monocopper oxidase-like protein SKU5 |
| AT5G25980 | 4.7 | Myrosinase |
| AT3G01500 | 4.5 | Beta carbonic anhydrase 1, chloroplastic |
| AT1G44350 | 4.5 | IAA-amino acid hydrolase |
| AT4G34710 | 4.4 | Arginine decarboxylase |
| AT1G77760 | 4.3 | Nitrate reductase |
| AT1G45201 | 4.1 | Triacylglycerol lipase-like 1 |
| **Downregulated genes** | | |
| AT2G33550 | −7.4 | Trihelix transcription factor ASR3 |
| AT1G66390 | −7.1 | Transcription factor MYB90 |
| AT1G21120 | −7.0 | O-methyltransferase family protein |
| AT2G28720 | −6.3 | Histone |
| AT1G56250 | −6.2 | F-box protein VBF |
| AT2G16600 | −6.0 | Peptidyl-prolyl cis-trans isomerase |
| AT5G23750 | −5.6 | Remorin family protein |
| AT3G52280 | −5.5 | General transcription factor group E6 |
| AT1G68790 | −5.4 | Protein CROWDED NUCLEI |
| AT2G40880 | −5.3 | Cysteine proteinase inhibitor |
| AT3G51920 | −5.1 | Calmodulin-like protein |
| AT5G05410 | −5.1 | Dehydration-responsive element-binding protein 2A |
| AT5G52740 | −5.1 | Heavy metal-associated isoprenylated plant protein12 |
| AT3G51910 | −5.0 | Heat stress transcription factor A-7a |
| AT5G16570 | −5.0 | Glutamine synthetase |
| AT5G16470 | −5.0 | Protein METHYLENE BLUE SENSITIVITY 2 |
| AT1G76720 | −4.9 | Eukaryotic translation initiation factor 2 (eIF-2) |
| AT5G40340 | −4.9 | Tudor/PWWP/MBT superfamily protein |
| AT1G22160 | −4.9 | FCS-Like Zinc finger 5 |
| AT5G39950 | −4.8 | Thioredoxin H2 |
| AT5G55660 | −4.3 | DEK domain-containing chromatin associated protein |
| AT5G52640 | −4.3 | Heat shock protein 90 |
| AT4G19840 | −4.3 | Protein PHLOEM PROTEIN 2-LIKE A1 |
| AT4G29160 | −4.2 | Vacuolar protein sorting-associated protein |
| AT3G25230 | −4.2 | Peptidylprolyl isomerase |
| AT3G52400 | −4.0 | Syntaxin |
| AT2G33550 | −7.4 | Trihelix transcription factor ASR3 |

**TABLE 3** Most important differentially expressed genes in *B.* *altitudinis* during the interaction with Arabidopsis plants

| **ID** | **Log2 Fold Change ^a^** | **Swissprot** |
| --- | --- | --- |
| **Upregulated genes** | | |
| gene2967 | 5.3 | MFS transporter |
| gene2185 | 4.5 | Sucrose-6-phosphate hydrolase |
| gene1412 | 4.0 | Phosphate ABC transporter |
| gene3573 | 3.5 | DNA gyrase subunit B |
| gene1888 | 3.3 | Polysaccharide biosynthesis protein |
| gene3614 | 3.3 | Serine phosphatase |
| gene273 | 3.3 | Flagellar biosynthesis protein |
| gene1747 | 2.7 | Electron transfer flavoprotein subunit beta |
| gene3085 | 2.5 | Phosphoribosylaminoimidazole synthetase |
| gene1805 | 2.3 | Bifunctional oligoribonuclease/PAP phosphatase NrnA |
| gene3086 | 2.3 | Phosphoribosylglycinamide formyltransferase |
| gene1799 | 2.2 | Acetyl-carboxylase subunit beta |
| gene1627 | 2.0 | Rod shape-determining protein |
| gene1555 | 1.7 | Uridine kinase |
| gene388 | 1.6 | Cell division protein |
| gene459 | 1.5 | Branched-chain alpha-keto acid dehydrogenase subunit E2 |
| gene2681 | 1.4 | 3-hydroxyacyl-CoA dehydrogenase |
| gene1634 | 1.2 | Valyl-tRNA synthetase |
| gene2803 | 1.1 | Alpha-mannosidase |
| gene369 | 1.0 | Isoleucine |
| gene284 | 1.0 | Flagellar basal body rod protein |
| gene389 | 1.0 | Cell division protein |
| **Downregulated genes** | | |
| gene3695 | −3.8 | Elongation factor G |
| gene1531 | −2.7 | 2,3-butanediol dehydrogenase |
| gene1048 | −2.6 | Dihydrolipoamide succinyltransferase succinyl transferase |
| gene2911 | −2.6 | Proline dehydrogenase |
| gene3548 | −1.9 | Single-stranded DNA-binding protein |
| gene668 | −1.7 | Flagellin |
| gene3183 | −1.4 | Crp/Fnr family transcriptional regulator |
| gene3475 | −1.2 | Flotillin |

^a^ Genes with a Log2 Fold Change between -1.2 and 5.3 were included.
